# Transcriptomic Changes of *Piscirickettsia salmonis* During Intracellular Growth in a Salmon Macrophage-Like Cell Line

**DOI:** 10.3389/fcimb.2019.00426

**Published:** 2020-01-09

**Authors:** Alejandro Zúñiga, Pamela Aravena, Rodrigo Pulgar, Dante Travisany, Javiera Ortiz-Severín, Francisco P. Chávez, Alejandro Maass, Mauricio González, Verónica Cambiazo

**Affiliations:** ^1^Laboratorio de Bioinformática y Expresión Génica, Instituto de Nutrición y Tecnología de los Alimentos (INTA), Universidad de Chile, Santiago, Chile; ^2^Blue Genomics Chile, Puerto Varas, Chile; ^3^FONDAP Center for Genome Regulation, Santiago, Chile; ^4^Center for Mathematical Modeling (PIA AFB17001) and Department of Mathematical Engineering, Universidad de Chile - UMI CNRS 2807, Santiago, Chile; ^5^Laboratorio de Microbiología de Sistemas, Facultad de Ciencias, Universidad de Chile, Santiago, Chile

**Keywords:** *Piscirickettsia salmonis*, stringent response, virulence factors, Piscirickettsiosis, SHK-1 cells, (p)ppGpp, type IVB secretion system

## Abstract

*Piscirickettsia salmonis* is the causative agent of Piscirickettsiosis, a systemic infection of salmonid fish species. *P. salmonis* infects and survives in its host cell, a process that correlates with the expression of virulence factors including components of the type IVB secretion system. To gain further insights into the cellular and molecular mechanism behind the adaptive response of *P. salmonis* during host infection, we established an *in vitro* model of infection using the SHK-1 cell line from Atlantic salmon head kidney. The results indicated that in comparison to uninfected SHK-1 cells, infection significantly decreased cell viability after 10 days along with a significant increment of *P. salmonis* genome equivalents. At that time, the intracellular bacteria were localized within a spacious cytoplasmic vacuole. By using a whole-genome microarray of *P. salmonis* LF-89, the transcriptome of this bacterium was examined during intracellular growth in the SHK-1 cell line and exponential growth in broth. Transcriptome analysis revealed a global shutdown of translation during *P. salmonis* intracellular growth and suggested an induction of the stringent response. Accordingly, key genes of the stringent response pathway were up-regulated during intracellular growth as well as at stationary phase bacteria, suggesting a role of the stringent response on bacterial virulence. Our results also reinforce the participation of the Dot/Icm type IVB secretion system during *P. salmonis* infection and reveals many unexplored genes with potential roles in the adaptation to intracellular growth. Finally, we proposed that intracellular *P. salmonis* alternates between a replicative phase and a stationary phase in which the stringent response is activated.

## Introduction

*Piscirickettsia salmonis*, the causative agent of Piscirickettsiosis, also known as Salmon Rickettsial Septicemia (SRS) was first identified as a pathogenic agent in disease outbreaks among farmed Coho salmon (*Oncorhynchus kisutch*). *P. salmonis* produces a systemic infection characterized by the colonization of several organs including kidney, liver, spleen, intestine, brain, ovary, and gills (Fryer et al., 1992). This bacterium was initially isolated in 1989 from a moribund coho salmon, during an epizootic event that took place in the south of Chile (Fryer et al., 1990; Branson and Nieto Diaz-Munoz, 1991; Cvitanich et al., 1991). Since then, *P. salmonis* infectivity has been demonstrated in all farmed salmonid fish species. *P. salmonis* covers a wide geographic range and outbreaks of Piscirickettsiosis have been reported among farmed salmonid in Canada, Norway, and Ireland; however, mortalities have not been as high as those recorded in Chile (Rozas and Enríquez, 2014).

Previous studies have analyzed the cellular interaction between *P. salmonis* and eukaryotic cells, especially the ability of this pathogen to survive within host cells. In an early study, McCarthy et al. (2008) using transmission electron microscopy showed that escape into the macrophage cytoplasm is not used to avoid lysosome fusion; instead, the bacterium remains at least partly enclosed within a vacuole membrane. In addition, there are evidences that *P. salmonis* can manipulate signaling pathways of the host cell. For instance, Rojas et al. (2010) showed that *P. salmonis* induces apoptosis in macrophages and monocyte-like cells; however, the mechanism behind this process remains to be elucidated. *P. salmonis* virulence factors are poorly characterized although the expression of four components of the type IVB secretion system during bacterial infection has been reported by Gómez et al. (2013). This is a major secretion system that can translocate virulence factors (effectors) into the host cell to subvert the host signaling pathways (Chandran Darbari and Waksman, 2015). Other reports have addressed different aspects of *P. salmonis* interaction with host immune cells (Isla et al., 2014; Ramírez et al., 2015; Salazar et al., 2015); however, further studies are required for unraveling the pathogenic mechanisms of *P. salmonis*.

Elucidation of the *P. salmonis* transcriptome during infection of its host cell can provide a better understanding of the *in vivo* process since genes expressed during infection can reveal which portions of the genome are tasked with promoting infection of host cells or facilitate pathogen survival in the macrophage environment. This approach has been used in the past to reveal virulence determinants of several intracellular bacteria, among them *Francisella tularensis* (Wehrly et al., 2009) and *Salmonella typhimurium* (Hautefort et al., 2008). The availability of the complete genome sequence for *P. salmonis* (Pulgar et al., 2015a) enabled us to design a whole genome DNA microarray to identify *P. salmonis* genes regulated during infection of macrophages. SHK-1, a head kidney cell line from Atlantic salmon with macrophage-like characteristics, was selected to establish an *in vitro P. salmonis* infection model. Cell viability and intracellular multiplication of *P. salmonis* along with the morphology of the infection were considered to select a late stage of *P. salmonis* infection for transcriptional analysis. As control, transcription of *P. salmonis* growing *in vitro* from exponential phase cultures was examined. The results of this study reinforces the participation of the Dot/Icm type IVB secretion system during *P. salmonis* infection, introduces a potential regulatory role of the stringent response pathway and the alarmone (p)ppGpp on virulence, and reveals that there are still many unexplored genes, which could be critical for intracellular growth of this bacterium. Overall, these data provide insights into genes involved in survival and adaptation of the bacterium within macrophage cells as well as a new understanding of the biology of host–pathogen interaction during *P. salmonis* infection. Thus, our results extended the number of putative virulence factors and processes associated to Piscirickettsiosis that could be considered in future antimicrobial strategies.

## Materials and Methods

### Cells and Bacteria Culture Conditions

*P. salmonis* LF-89 (ATCC VR-1361) used in this study was obtained from the American Type Culture Collection (ATCC). The bacteria were routinely maintained by sub-culturing in agar and liquid broth (AUSTRAL-SRS, Yañez et al., 2013) with agitation (180 rpm) at 18°C. Each subculture was confirmed as *P. salmonis* by Gram staining and RFLP assay (Mandakovic et al., 2016). In our conditions, *P. salmonis* reach the mid-exponential phase 1.5 days and the stationary phase 3 days post-inoculation into fresh medium (Supplementary Figure 1). In order to obtain bacterial cultures with 1, 2, 3, and 4 days of growth, every day for 4 days, bacteria grown on agar plates were inoculated into Austral-SRS broth. SHK-1 cells were obtained from European Collection of Authenticated Cell Cultures (ECACC) and were cultivated at 18°C in Leibovitz's L-15 Medium (Gibco, USA) supplemented with 5% of inactivated fetal bovine serum and 40 μM of 2-mercaptoethanol.

### *In vitro* Infection

Stationary phase bacteria were obtained by growing *P. salmonis* LF-89 in 5 ml of nutrient broth (AUSTRAL-SRS) in 50-ml falcon tubes for 4 days at 18°C and 180 rpm (orbital shaker LabTech). After checking bacteria purity, OD_600_ was measured and correlated with bacterial cell numbers counting using a Petroff–Hausser chamber. Cell viability was evaluated with the most-probable number (MPN) method (Sutton, 2010). The results are shown in Supplementary Table 1. SHK-1 cells were seeded at 4 × 10^5^ cells per T-25 flask in Leibovitz's L-15 medium supplemented with 5% of fetal bovine serum (FBS) and without antibiotics. Near-confluent cells (80%) were infected with stationary phase bacteria at a multiplicity of infection (MOI) of 100:1 (bacteria:cells) at 18°C. Three days post-infection (dpi), cells were washed with PBS and then incubated for 40 min with L-15 medium plus gentamicin (100 μg/ml) to kill extracellular bacteria. After incubation, cells were washed with PBS and incubated in L-15 medium supplemented with 5% of FBS. Cells were observed under optical inverted microscope to follow the progression of the infection.

### Immunofluorescence Assays

SHK-1 cells were seeded on coverslips and infected as was described above. Before fixation, cells were treated for 30 min with 2.5 mg/ml DiI (1,1′-dioctadecyl-3,3,3′,3′-tetramethylindocarbocyanine perchlorate, Thermo Fisher Scientific) to stain cell membranes. Then, cells were fixed with 4% paraformaldehyde for 10 min, permeabilized with 0.1% saponin in PBS, and blocked with 0.1% saponin in PBS plus 3% BSA for 30 min. Intracellular *P. salmonis* was detected by incubation with specific antibodies anti-*P. salmonis* (Ango, 1:200 dilution) for 1 h at room temperature. After several washes with PBS, cells were incubated with secondary anti-mouse FITC-conjugated antibody (1:200 dilution) for 1 h and with the following fluorescent probes: phalloidin-Alexa 636 (Thermo Fisher Scientific, 1:200 dilution) for 15 min to detect polymerized actin and DAPI (4′,6-diamidino-2-phenylindole, Thermo Fisher Scientific, 1:200 dilution) for 15 min to detect DNA. Images were acquired in a C2+ Confocal microscope (Nikon) using NIS-elements program (Nikon).

### *P. salmonis* Replication in SHK-1 Cells

*P. salmonis* replication was determined using quantitative PCR (qPCR) of genome equivalents. SHK-1 cells were infected as described above and samples were collected for DNA analysis at 3, 7, and 10 dpi. At the different times post-infection, cell monolayers were washed with PBS and treated with 0.25% trypsin–EDTA solution. Cells were recovered by centrifugation at 12,000 × *g* for 10 min and the pellets were resuspended in 200 μl of PBS. Genomic DNA (gDNA) was isolated from three independent cultures of infected cells using the DNeasy Blood & Tissue kit (Qiagen), according to manufacturer instructions. The concentration and the quality of the DNA (absorbance 260/280 nm) in each sample were measured using a NanoQuant Spectrophotometer (Tecan Technologies); integrity was examined using the 2200 TapeStation Bioanalyzer (Agilent Technologies) and then gDNA samples were adjusted to a concentration of 15 ng/μl. *P. salmonis* genome equivalents in infected SHK-1 cell samples were estimated using qPCR and the Forget-Me-Not Evagreen qPCR Master Mix kit in an AriaMx Pro (Agilent Technologies) real-time cycler, with primers that target the *P. salmonis glyA* gene (GenBank accession NZ_CP011849.2). Briefly, the reaction mixture contained 10 μM of each primer, and 30 ng of sample template DNA in a total volume of 25 μl. The following PCR conditions were used: initial denaturation at 95°C for 2 min, 40 cycles of 95°C for 5 s, 61°C for 10 s, and 72°C for 15 s, with a melt step of 95°C for 30 s, 65°C for 30 s, and 95°C for 30 s. The resulting fluorescent plots were analyzed, and estimated numbers of *P. salmonis* genomes in the experimental samples were determined based on a calibration curve. The calibration curve was developed from PCR products of the single-copy gene *glyA*. The PCR product (133 bp) was purified using Wizard® SV Gel and PCR Clean-Up System Kit (Promega) and product concentration was measured by Qubit fluorometric quantitation. Serially diluted PCR product (10^1^ to 10^6^ copies) was used as a template to generate standard curves for qPCR assays. The conversion from copy number to genome equivalent was based on the presence of only one copy of *glyA* gene in the *P. salmonis* genome. Three biological replicates with three technical replicates per sample were used in the analysis of each time point. Data were analyzed with GraphPad Prism V8.0.1 using an unpaired *t*-test with Welch‘s correction. Means ± SD were reported.

### Cell Viability/Cytotoxicity Assays

Cell viability was quantified using the non-toxic colorimetric indicator alamarBlue™ (Thermo Fisher Scientific). SHK-1 cells were seeded into four 96-well plates (1 × 10^4^ cells/well) with Leibovitz's L-15 medium pH 7.0 supplemented with 5% FBS and incubated at 18°C for 24 h. Then, for each plate, 12 wells were infected with *P. salmonis*, following the procedures described above, and 12 wells were left uninfected. After incubating for 3 days with the bacteria, infected and uninfected cells were washed with PBS and then incubated for 40 min with L-15 medium plus gentamicin (100 μg/ml); after incubation, cells were washed with PBS and incubated in L-15 medium supplemented with 5% of FBS. Cells were observed under an optical microscope to follow the progression of the infection, and at 3, 7, 10, and 12 dpi, one plate was processed for alamarBlue quantification. To do that, the medium was discarded, and fresh L-15 medium containing 50 μg/ml of gentamicin, 300 U/ml of penicillin/streptomycin, 5% SFB, and 10% of the alamarBlue reagent was added to the wells. Absorbance of the plate was measured at 570 and 600 nm in an Infinite® 200 PRO NanoQuant (Tecan®) at 10 h after adding the reagent. Percentages of alamarBlue reagent reduction were calculated with respect to the control cells (uninfected) following the formula (Al-Nasiry et al., 2007):

$$\begin{array}{l} \frac{\left(\varepsilon_{OX}\right)\lambda_{2}\;A\lambda_{1}-\left(\varepsilon_{OX}\right)\lambda_{1}\;A\lambda_{2}\;infected\;cells}{\left(\varepsilon_{OX}\right)\lambda_{2}\;\text{A}{}^{\circ}\lambda_{1}-\left(\varepsilon_{OX}\right)\lambda_{1}\;\text{A}{}^{\circ}\lambda_{2}\;uninfected\;cells}\times \;100 \end{array}$$

where εOX = 0.6633 molar extinction coefficient of oxidized AB

A = Absorbance infected cells

A° = Absorbance control cells

λ_1_ = 570 nm; λ_2_ = 600 nm

A similar alamarBlue assay was carried out, but this time, the percentages of alamarBlue reduction were calculated with respect to cells at the beginning of the assay. Briefly, SHK-1 cells were seeded into 96-well plates (*N* = 10 wells per treatment) and infected with *P. salmonis* or left uninfected. Afterwards, the cultures were washed and the bacteria were removed (day 3). Cell viability was monitored over time, and at 3, 7, 10, and 12 dpi, one plate was processed for alamarBlue quantification as previously described. Additionally, cell viability was assessed using the Viability/Cytotoxicity Assay Kit for Animal Live & Dead Cells (Biotium) according to the manufacturer's instructions. Briefly, monolayers of *P. salmonis*-infected SHK-1 and uninfected SHK-1 cells were rinsed with PBS twice. Then, a mix of 2 μM calcein-AM and 4 μM ethidium homodimer-III was added to the cell monolayer and cells were incubated for 45 min at room temperature. Images were acquired in a C2+ Confocal microscope (Nikon) using NIS-elements program (Nikon) and cell counting was done manually.

To address the decrease in cell viability when cells were infected with bacteria at exponential or stationary state of growth, SHK-1 cells were seeded into four 96-well plates and infected with *P. salmonis*, previously grown for 24, 48, 72, or 96 h in liquid broth, or left uninfected (*N* = 12 wells per treatment). After incubating for 3 days with the bacteria, infected and uninfected cells were washed and incubated in L-15 medium supplemented with 5% of FBS for 10 days. Afterwards, cells were processed for alamarBlue quantification as described above. Viability of *P. salmonis*-infected cells was compared with that of uninfected cells and expressed as a percentage of cytotoxicity, using the following formula:

$$\begin{array}{l} 1-(\text{mean absorbance of infectedcells}\text{​​}/\\ \;\text{mean absorbance of untreated cells})\;\times \;100 \end{array}$$

### RNA Extraction From *P. salmonis* Cultures

To isolate total RNA from exponential or stationary phase *P. salmonis* cultures, bacteria were collected from the broth by centrifugation, resuspended in 0.5 ml of RNAWIZ (Ambion), mixed with 200 μl of zirconia beads, and disrupted using the Fast-prep 24 (MP) in three cycles of 40 s at 6.0 M/s with intervals of 5 min on ice. The bacterial lysate was recovered by centrifugation and zirconia beads were discarded. The bacterial lysate was mixed with 0.2 volumes of chloroform and then centrifuged. The aqueous phase was mixed with 0.5 volumes of 100% ethanol and bacterial RNA was purified by passing through a filter cartridge (Qiagen); successive washes were carried out according to manufacturer's instructions. RNA was eluted with 0.1 ml of TE buffer in DEPC-treated water and incubated for 30 min at 37°C with RNase-Free DNase I (Ambion) to remove residual gDNA. The quantity and quality of RNA were determined by measuring the absorbance at 260/280 nm using a NanoQuant Spectrophotometer (Tecan Technologies) and integrity was confirmed using 2200 TapeStation Bioanalyzer (Agilent Technologies).

### RNA Extraction From *P. salmonis*-Infected SHK-1 Cells

Monolayers of infected SHK-1 cells were rinsed with PBS and resuspended in 1.0 ml of RNAWIZ (Ambion), and total RNA was purified using the RiboPure-Bacteria kit (Ambion) following manufacturer's instructions. The fraction of intracellular *P. salmonis* RNA was enriched by subtracting eukaryotic ribosomal and messenger RNA using the MICROBEnrich™ Kit (Life Technologies). Briefly, 25 μg of total RNA was mixed with 10 μl of oligocapture in 300 μl of binding buffer. The mixes were incubated at 70°C by 10 min, and at 37°C by 1 h. Then, magnetic oligo-MagBeads were added and incubated at 37°C by 15 min, the beads were captured by using a magnetic stand, and the supernatant containing bacterial enriched RNA was recovered. The recovered RNA was precipitated with sodium acetate, ethanol 100%, and glycogen and incubated at −20°C overnight. The pellets were centrifuged at 4°C, washed twice with cold 70% ethanol, and resuspended in 40 μl of TE buffer. The quantity and quality of RNA were determined by NanoQuant Spectrophotometer (Tecan Technologies) and 2200 TapeStation Bioanalyzer (Agilent Technologies).

### Microarray Design

In order to analyze *P. salmonis* gene expression, we designed a pathogen-specific oligo microarray using the eArray system (Agilent Technologies). *P. salmonis* LF-89 gene targets were derived from the genome assembly and annotation was reported in Pulgar et al. (2015b). Each array contained 15,208 60-mer oligonucleotides (5310 different probes) representing 2850 *P. salmonis* gene targets with at least two duplicate probes per gene. The array also contained positive and negative controls, previously selected by Agilent for use in commercial microarrays. EST sequences of *Salmo salar* were used as queries in the design step to reduce the likelihood of designing probes that would cross-hybridize with host sequences. Selected probes were synthesized *in situ* on a glass slide in an 8 × 15 K format using Agilent SurePrint technology. Specificity and accuracy of the microarray for bacterial transcripts were assessed by performing hybridizations with gDNA from SHK-1 and from *P. salmonis* (data not shown). gDNA was isolated using the DNeasy Blood & Tissue kit (Qiagen) from T-25 flasks of confluent SHK-1 cells or 1 ml of exponential phase *P. salmonis* culture, according to the manufacturer's instructions.

### Labeling of RNA and DNA Samples

One hundred nanograms of RNA isolated from exponentially growing *P. salmonis* or from *P. salmonis*-infected SHK-1 cells were labeled using Low Input Quick Amp kit (Agilent) following the manufacturer's instructions. The resulting cRNA with incorporated CNTP-Cy3 or CNTP-Cy5 was purified using RNeasy Kit (Qiagen). The quantity and quality of cRNA and label incorporation were determined by the NanoQuant Spectrophotometer (Tecan Technologies). gDNA (20 ng) from SHK-1 or *P. salmonis* was separately labeled using SureTag complete DNA labeling kit (Agilent) according to the manufacturer's instructions.

### Microarray Hybridization

Cy3- and Cy5-labeled RNA samples were hybridized in the 8 × 15 K slides using the Agilent Gene Expression Hybridization Kit. Briefly, 300 ng of each amplified and labeled samples were mixed with blocking agent and fragmentation buffer, incubated 30 min at 60°C, followed by 1 min on ice, and then 2× hybridization buffer was added. The mix was added onto the slide and incubated in a hybridization oven at 65°C for 17 h. Then, slides were sequentially rinsed with GE Wash Buffer 1 at room temperature for 1 min and then with GE Wash Buffer 2 for 1 min at 37°C. Finally, slides were dried at room temperature.

### Microarray Data Analysis

Microarrays slides were scanned using the Microarray Scanner (G2565CA) from Agilent Technologies at 5 μm resolution and at high and low photo multiplier voltages to optimize the dynamic range of the image. Data were extracted from images using the Agilent Feature Extraction 10.7.3.1 software (Agilent Technologies) with default protocols and settings. Quality of microarray images was examined by inspection of foreground (FG) and background (BG) values of red (R) and green (G) channels in each slide. Data were background corrected using a normexp procedure (Ritchie et al., 2007) and normalized between channels using an intensity-dependent normalization procedure (loess) (Smyth and Speed, 2003). Linear models were fitted to log_2_ expression data for each gene across the microarrays (Yang et al., 2002). In each case, the coefficient of the model represented the estimated fold change between samples. These values were used to compute a moderated t-statistics using a simple Bayesian model and raw *p* values were corrected for multiple testing using a false discovery rate (FDR) of 0.05. Genes with FDR < 0.05 were acknowledged as differentially expressed (DEGs). All calculations were performed on R statistical software environment running on Linux or Windows machine, using limma package (Smyth, 2005; Pulgar et al., 2015a). Microarray data were submitted to Gene Expression Omnibus (Accession number: GSE128825).

### Bioinformatics Analysis

The functional annotation was performed using BLASTp against NR, UniProtKB, and KEGG. InterproScan was used for the identification of conserved domains (Quevillon et al., 2005). Virulence factors for each isolate were predicted using the complete set of proteins from VFDB (VFDB_setB.pro.fas). The Blast2GO software was used to assign descriptions of gene function and also gene ontology categories (Conesa et al., 2005). The entire set of predicted protein sequences from *P. salmonis* LF-89 were blasted against the GenBank non-redundant protein database with results filtered for E < 10^−5^, and also queried against the InterPro functional prediction pipeline. Functional enrichment of Gene Ontology (GO) terms present in the DEGs relative to the genomic background was performed using Blast2GO's implementation of a Fisher's exact test with a *p* < 0.01 (Gotz et al., 2008). Amino acid sequence similarities were obtained using the TaxonDC software (Tarlachkov and Starodumova, 2017).

### Quantitative Real-Time PCR Assays (qPCR)

Reactions were carried out in an Mx3005P System (Stratagene) using the LightCycler-FastStart DNA Master SYBR Green kit (Roche Applied Science) and MxPro QPCR software (Stratagene). In all cases, cDNAs were synthesized from 2 μg of RNA using the High-Capacity RNA-to-cDNA Kit (Applied Biosystems) according to the manufacturer's instructions. cDNAs were diluted to 100 ng and used as template for qPCR, with primers designed against genes of interest. PCR conditions were 95°C for 5 min followed by 94°C for 15 s, 57–60°C for 15 s, and 72°C for 20 s for a total of 35 cycles. Melting curves (1°C steps between 75 and 95°C) ensured that a single product was amplified in each reaction. To determine relative expression levels of genes, the method described by Pfaffl (2001) was employed, using gene *glyA* as an internal reference gene. At least three biological replicates with three technical replicates each were analyzed and PCR efficiencies were determined by linear regression analysis performed directly on the sample data using LinRegPCR (Ramakers et al., 2003). Supplementary Table 2 shows the complete list of primers used in this study.

To validate the results from the microarray experiments, 65 genes were selected from the microarray data and their expression levels were determined by qPCR (list of genes and primer sequences in Supplementary Table 2). For each gene, mean log_2_ ratios (infected/control) determined by microarrays were plotted against mean log_2_ ratios (infected/control) determined by qPCR assays. Correlations between microarrays and qPCR were calculated by Pearson correlation and a *p* < 0.01 was considered as statistically significant.

## Results and Discussion

### Infection Model

Considering that transcriptional changes associated to *in vivo* infection with *P. salmonis* could be affected by environmental and physiological features of different cellular types in the host, we aimed to establish an *in vitro P. salmonis* infection model suitable for the study of host–pathogen interactions. With this purpose, the SHK-1 cell line derived from Atlantic salmon (*S. salar*) macrophages (Dannevig et al., 1997) was selected because it represents a cell type that is infected by this bacterium *in vivo* (McCarthy et al., 2008; Rojas et al., 2009). The infection assays were carried out with *P. salmonis* LF-89, a strain whose circular chromosome and three plasmids represent the first complete genome sequenced of this bacterium (Pulgar et al., 2015b).

**Figure 1:**
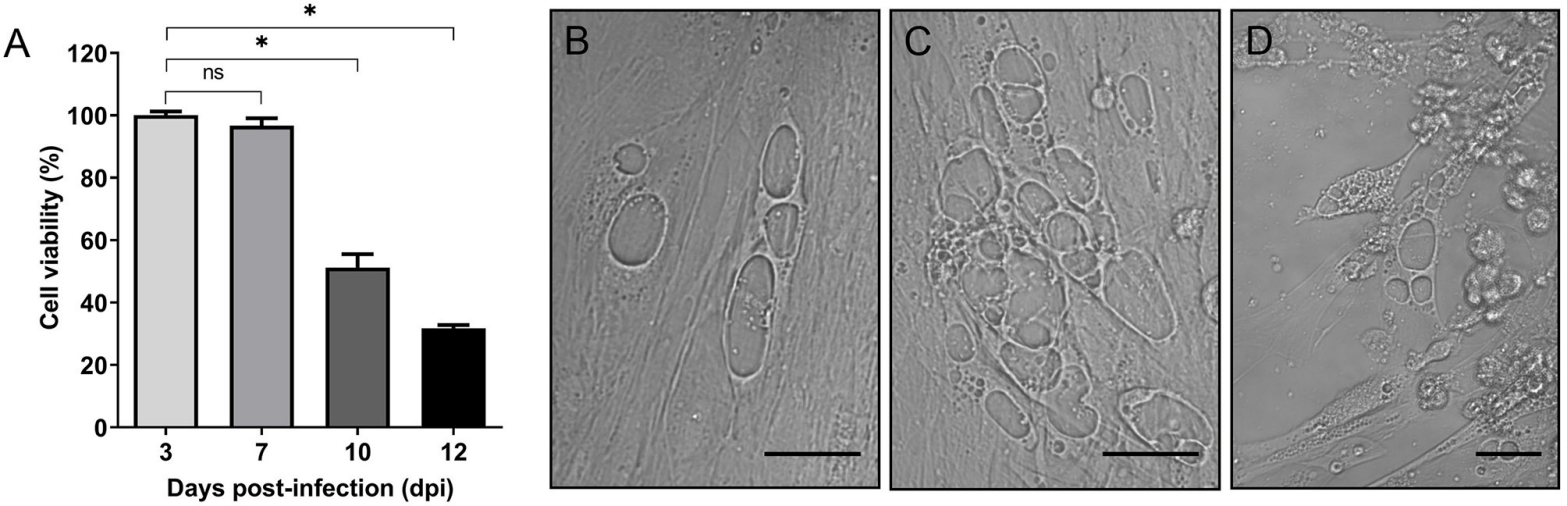
*In vitro* infection of SHK-1 cells. **(A)** Cells were infected with *P. salmonis* at a multiplicity of infection (MOI) of 100. Cell viability was analyzed by alamarBlue assay at different days post-infection (dpi). Data reflect means ± SD (*N* = 12 biological replicates); asterisks above the bars indicate significant differences (*p* < 0.05), ns, no significant differences. Data were analyzed with GraphPad Prism V8.0.1 using an unpaired *t*-test with Welch's correction. **(B–D)** Representative images of bright-field microscopy of SHK-1 cells at different days post-infection. **(B)** 7 dpi, **(C)** 10 dpi, **(D)** 12 dpi. Bar = 10 μm.

Given that cytopathogenicity of stationary phase *P. salmonis* to SHK-1 cells was significantly higher (>60%) when compared to exponential phase bacteria (Supplementary Figure 2), bacteria grown for 4 days were used for the infection assays. To assess the effect of *P. salmonis* infection on the viability of SHK-1 cells, the alamarBlue reagent was used in a colorimetric microplate assay. SHK-1 cells were infected for 3 days with stationary phase *P. salmonis* LF-89 (MOI of 100) and then washed and incubated with fresh L-15 medium plus gentamicin to kill extracellular bacteria. In different days post-infection (3, 7, 10, and 12 dpi), the viability of infected and uninfected cells was determined. The results revealed that *P. salmonis* infection process was associated with a significant decrease of cell viability at 10 and 12 dpi (*p* < 0.05) with respect to the percentage of viability measured at 3 or 7 dpi (Figure 1A). During this period, an increase of vesicles containing *P. salmonis* was observed by light field microscopy, suggesting that the presence of bacterium affected viability of SHK-1 cells (Figures 1B-D). Similarly, when cell viability was expressed as a percentage of the viability of cell cultures at the beginning of the infection assay, the results indicated that the decrease in viability of the SHK-1 cells infected with *P. salmonis* reached approximately 30% of control's viability at 12 dpi (Supplementary Figure 3). In agreement with the results of alamarBlue assays, measuring of cell viability using calcein-AM (which stains live cells) and ethidium homodimer-III (which stains dead cells), revealed significantly more death, at 12 dpi, in the infected cells with respect to control (uninfected) cells (Supplementary Figure 4).

**Figure 2:**
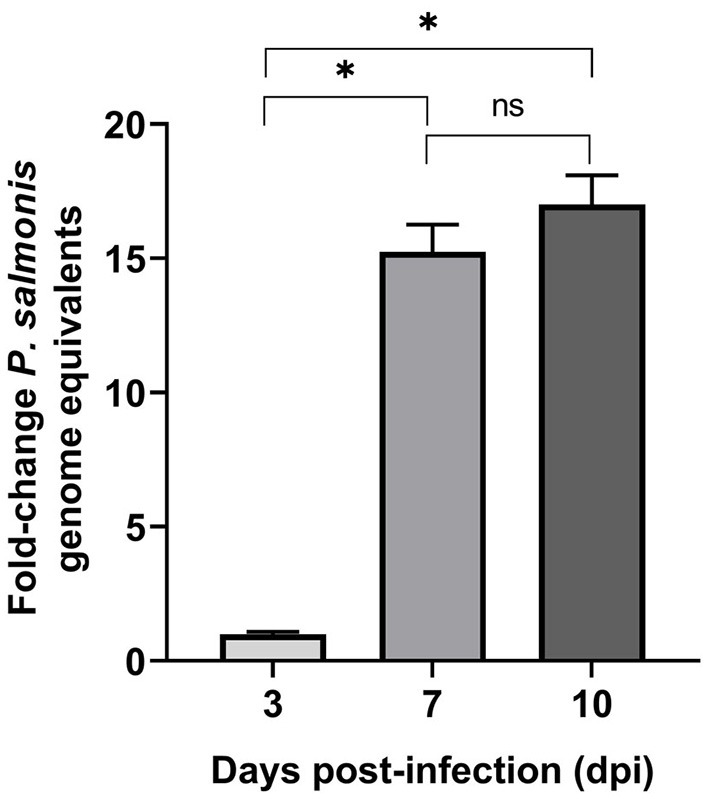
*P. salmonis* genome levels during infection of SHK-1 cells. For each sample, an equal amount of total gDNA was analyzed by quantitative qPCR with primers designed for the *glyA* gene. Results represent the mean of three biological samples with three technical replicates of each sample. Data are presented as fold changes in genome numbers relative to 3 dpi. Standard error bars represent the combined standard error of the mean per time point. Asterisks above the bars indicate significant differences (*p* < 0.05), ns, no significant differences. Data were analyzed with GraphPad Prism V8.0.1 using an unpaired *t*-test with Welch‘s correction.

Intracellular multiplication of *P. salmonis* in SHK-1 cells was assessed during the course of infection using qPCR. At 3, 7, and 10 dpi, infected cells were harvested, bacterial DNA was isolated, and genome equivalents indicative of intracellular microorganisms were determined by using primers designed to target the *P. salmonis glyA* gene. The result supported the presence of *P. salmonis* in SHK-1 cells and revealed a significant increase in *P. salmonis* genome equivalents between 3 and 7 dpi (*p* < 0.05, Figure 2). *P. salmonis* genome equivalents were also increased between 7 and 10 days PI, although the increment was not statistically significant. Using the number of *P. salmonis* genomes equivalents at 3 dpi as a base line, a 15-fold and 17-fold of increment was detected at 7 and 10 dpi, respectively. Thus, the results indicated that *P. salmonis* productively infected and replicated in SHK-1 cells and suggested that the increment in the number of intracellular bacteria (15-fold) irreversibly disrupted cell integrity, affecting its viability.

**Figure 3:**
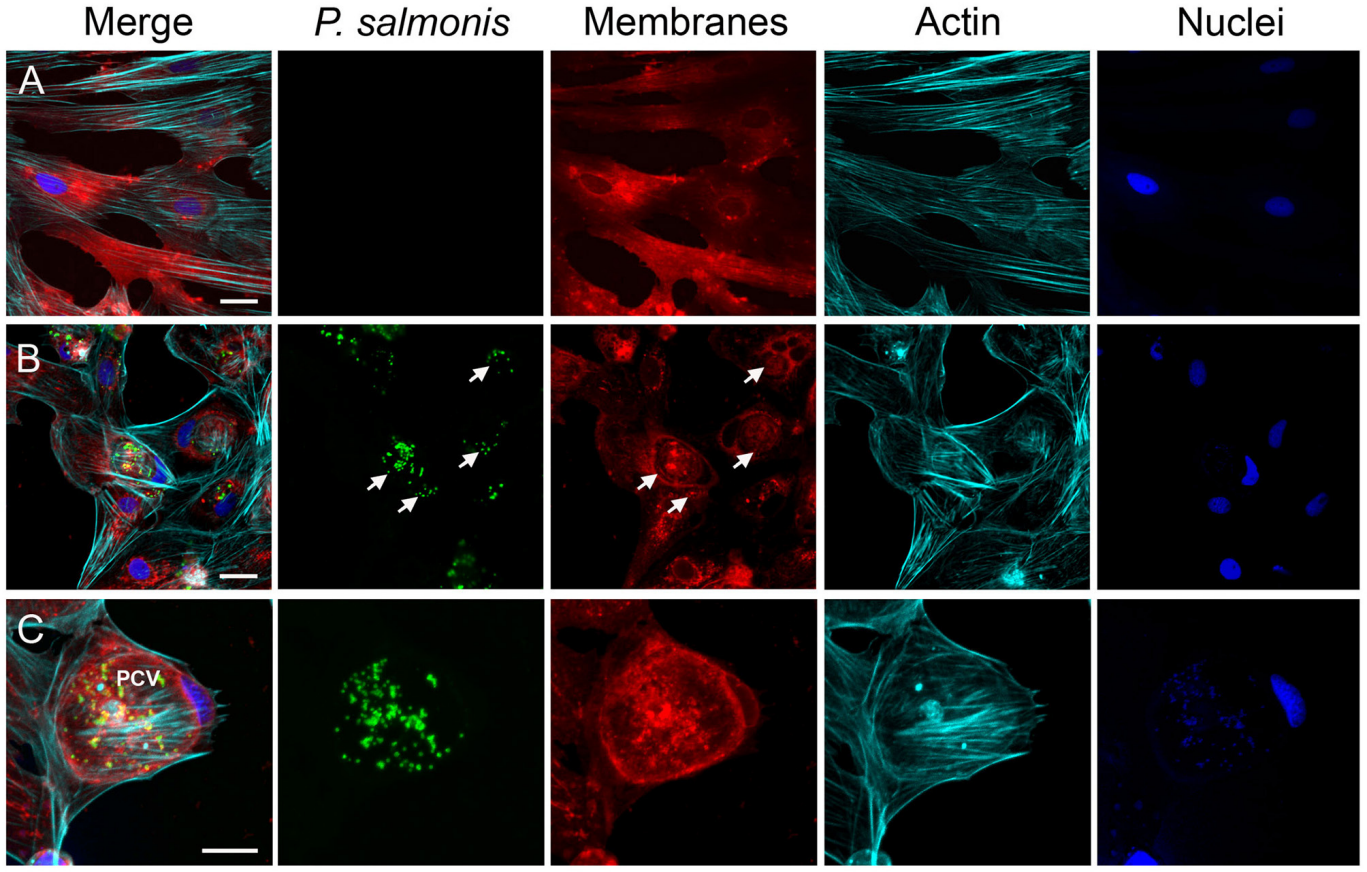
Characterization of the infection process by immunofluorescence. Representative confocal laser scanning microscopy images of indirect immunofluorescence against *P. salmonis* using specific antibodies (green). SHK-1 cell organelles were stained with Fluor-546 DiI to detect cell membranes (red), Alexa Fluor-647 Phalloidin to detect polymerized actin (cyan), and DAPI to detect DNA (blue) as indicated at each panel. **(A)** Uninfected SHK-1 cells. **(B)** Infected SHK-1 at 10 dpi. The arrows indicate *P. salmonis* vacuoles. Bar = 20 μm. **(C)** Closer image of a *P. salmonis* vacuole. PCV: *P. salmonis*-containing vacuole. Bar = 10 μm.

To further examine *P. salmonis in vitro* infection, the cell membranes of uninfected (Figure 3A) and infected SHK-1 were labeled with DiI (red) and then fixed and stained with antibodies specific for *P. salmonis* (green); in addition, actin cytoskeleton was labeled with Alexa Fluor-phalloidine (cyan) and the DNA was labeled with DAPI (blue). At 10 dpi, confocal microscope images revealed that bacteria were localized in a large vacuole within the host cell cytoplasm, which, in turn, appears to displace the nucleus to one side of the cell, around the vacuole a meshwork of F-actin was assembled (Figure 3B). One striking feature was the large size of the *P. salmonis*-containing vacuole (PCV, Figure 3C). At this time, the average diameter of the vacuoles was >20 μm and contained a variable number of bacteria (Figure 3C). These observations indicate that *P. salmonis* resides and proliferates mainly within a spacious vacuole of SHK-1 cells, and suggest that the bacterium can manipulate the host cellular pathways to increase the vacuole size. A similar morphological pattern has been described during the infection process of *P. salmonis* in rainbow trout macrophages (McCarthy et al., 2008), suggesting that the bacterium strategy could not be affected by species-specific differences. Interestingly, several studies have shown that intracellular pathogens, such as *Coxiella* and *Chlamydia*, subvert the endomembrane system of the host by inducing a series of fusion events with different subcellular compartments to form a single large vesicle that support intracellular replication (Coleman et al., 2004; Di Russo Case et al., 2016). In fact, successful vacuolar-replicating bacterial pathogens have evolved strategies to hijack the endomembrane system of host cells, sabotage their signal transduction pathways, and evade antimicrobial responses in order to develop a suitable replicative vacuole (Asrat et al., 2014; Weber and Faris, 2018). A common feature of intracellular pathogens is the expression of one or more secretion systems to deliver multiple proteins, referred to as effectors, into host cells. These secretion systems are required to modulate host membrane trafficking and establish an intracellular replicative niche (Green and Mecsas, 2016). The genome sequence of different strains of *P. salmonis* indicates the presence of two or three of *dot*<*underline/*>*icm* gene clusters encoding the type IVB secretion system (Bohle et al., 2014; Pulgar et al., 2015b). *P. salmonis* Dot/Icm is homologous to the T4BSS of the Gram negative intracellular pathogens *Legionella pneumophila* and *Coxiella burnetii* (Gómez et al., 2013; Cortés et al., 2017). For these two intracellular pathogens, it has been demonstrated that effector proteins translocated by the T4BSS modulate host cell pathways to enable their intracellular replication and survival in the host cell. In the case of *P. salmonis*, the transcripts and proteins of several structural components of the T4BSS have been detected in *P. salmonis*-infected cells (Gómez et al., 2013; Cortés et al., 2017). Moreover, a mutation in a structural component of *P. salmonis* T4BSS leads to a reduced virulence *in vitro* and a decreased mortality in an *in vivo* assay (Mancilla et al., 2018), suggesting that the Dot/Icm system may be essential for *P. salmonis* intracellular replication and survival. It is expected that a deeper understanding of how *P. salmonis* manipulates host membrane fusion pathways will provide mechanistic insight into how infection occurs and ultimately inspire novel therapeutic strategies to combat this infection.

### General Overview of the Transcriptional Profile of Intracellular *P. salmonis*

In this work, we aimed to further explore the cellular mechanisms that underlie the adaptive response of *P. salmonis* during infection in a macrophage-like cell line by comparing intracellular bacteria transcriptome to that of bacteria growing exponentially in liquid cultures. The survival of *P. salmonis* in the *in vitro* SHK-1 model of infection could be due to multiple physiological changes in the bacteria, aimed to promote their proliferation within the host cells. These changes could be inferred through a comparative transcriptome analysis (Rohde et al., 2012).

The transcriptional profile of intracellular *P. salmonis* was determined using a whole-genome microarray. This strategy has shown a good performance when compared with other methods of transcriptome analysis (Nookaew et al., 2012), and it has been successfully used to identify the transcriptional response of intracellular pathogen during an infection process (Eriksson et al., 2002; Belland et al., 2003; Mäurer et al., 2007; Abhishek et al., 2018). Our microarray contained 5310 different probes targeting 2850 genes that corresponded to 82% of the predicted coding sequences of *P. salmonis* strain LF-89. RNA from *P. salmonis* and SHK-1 cells were extracted simultaneously to avoid changes in transcriptomic profile after the bacterial purification process. Eukaryotic RNA was depleted using the MicrobEnrich Kit in order to increase the representation of bacterial transcriptome (La et al., 2007; Westermann et al., 2017), a strategy recently applied to analyze the transcriptional profile of *P. salmonis* from head kidney and spleen tissues of Atlantic salmons infected *in vivo* with the bacterium (Valenzuela-Miranda and Gallardo-Escárate, 2018). Based on the results shown above, we selected 10 dpi as the time point to extract RNA from *P. salmonis*-infected cells, ensuring the recovery of enough intracellular *P. salmonis* from still viable cells. In our experimental conditions, at least 60% of SHK-1 cells were infected with *P. salmonis* LF-89 at a MOI of 100. For each condition studied, three independent biological replicates were analyzed. Statistical analysis indicated that, in comparison with *P. salmonis* grown in liquid culture, 963 genes were differentially expressed (DEGs) at 10 dpi (FDR < 0.05). Of these, 494 genes demonstrated increased expression while 469 genes were down-regulated (Supplementary Table 3).

**Figure 4:**
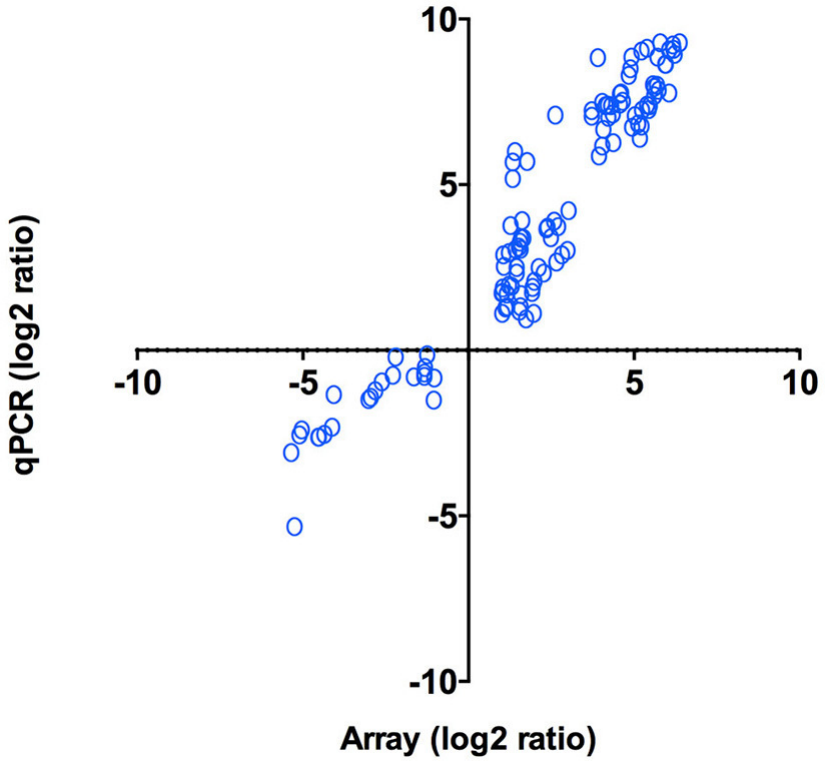
qPCR validation of microarray results. Log_2_ ratios (infected/control) of gene expression (*N* = 47) calculated from microarrays were plotted against the log_2_ ratios derived from qPCR assays. Correlation between microarrays and qPCR was calculated using Pearson correlation (*p* < 0.01).

As an independent measure of differential gene expression, we examined the relative expression of 65 genes selected from different functional categories by real-time qPCR (Figure 4) on the same RNA samples as those used for microarray hybridization assays. Among the 65 genes that were subjected to validation, 47 (72%) displayed the same trend observed in the microarray analysis (Supplementary Table 2). Overall, a positive correlation of 0.95 (Pearson correlation) was determined between microarray and qPCR for the combined data set (*p* < 0.01). The strong correlation observed verified the efficiency and robustness of the designed microarray for high-throughput screening of intracellular *P. salmonis* transcriptome.

### Intracellular Growth of *P. salmonis* Induces the Stringent Response

We performed a gene function enrichment analysis on DEGs to categorize them into biological processes. For simplicity, we use the terms up- and down-regulation to describe the relative differences between intracellular and exponentially growing *P. salmonis*; however, further analyses are necessary to dissect the transcriptional mechanism(s) that underlie these gene expression changes. The group of up-regulated genes relates to a diverse set of functions, such as oxidation–reduction process, transmembrane transport, phosphorylation, and gene expression; however, no significant enriched GO terms were detected. On the other hand, the analysis of significantly enriched functions of down-regulated genes (Supplementary Figure 5) suggested that the stringent response (Chatterji and Ojha, 2001) was induced during *P. salmonis* intracellular growth. Accordingly, genes down-regulated during intracellular growth included 50 genes involved in protein biosynthesis, such as those encoding ribosomal proteins, elongation factors, ribosome biogenesis and ribosome maturation factors, and tRNA modification enzymes (Supplementary Table 4). Down-regulation of genes involved in translation is consistent with the stringent response elicited among bacteria during unfavorable growth conditions (Potrykus and Cashel, 2008; Srivatsan and Wang, 2008) including restrictive conditions imposed by the host cells and nutrient repletion in the stationary phase (Prusa et al., 2018). The stringent response is a set of physiological changes that occur in bacteria in response to different environmental stress, and it is mediated by the synthesis and accumulation of the alarmones guanosine tetraphosphate (ppGpp) and guanosine pentaphosphate (pppGpp), which are collectively known as (p)ppGpp. As demonstrated for other pathogens, the intracellular growth of *P. salmonis* should involve bacterial adaptation to specific physical conditions such as O_2_ concentration, temperature, pH, and also to a changing nutritional environment within host cells (Kumar and Valdivia, 2009; Pulgar et al., 2015a; Prusa et al., 2018). Since the number of bacteria contained in the vacuole significantly increased at 10 dpi, we speculate that *P. salmonis* could be faced with intra-vacuolar nutrient limitation along with a harsh intracellular environment at a late phase of growth, and the ability to adapt to such condition could be defined as the *P. salmonis* stringent response.

As already known from *E. coli*, the hallmark of the stringent response consists of the negative regulation of components of the translational apparatus including rRNAs, tRNAs, ribosomal proteins, and translation factors (Traxler et al., 2008). Accordingly, it has been proposed for *E. coli* that the effector molecule (p)ppGpp binds to the β′-subunit of RNA polymerase (Hauryliuk et al., 2015; Ross et al., 2016), causing a rapid reduction of the transcription of *rrn* operons, probably by reducing the stability of the open promoter–RNA polymerase complexes at *rrn* promoters. Thus, binding of (p)ppGpp to the RNA polymerase modifies the enzyme preference for alternative sigma factors and promoters and consequently changes gene expression profiles. Data concerning the transcriptional regulation of operons by (p)ppGpp in *P. salmonis* is completely unknown. However, this study shows that transcription of several genes encoding ribosomal proteins were switched off 10 dpi (35 out of 52 encoded in *P. salmonis* genome). Components of the transcription and replication processes were also down-regulated in the intracellular *P. salmonis*, including DNA polymerase III (δ and ε subunits), DNA gyrase/topoisomerase (A and B subunits), the δ and Ω subunits of RNA polymerase, and the sigma factor RpoD. Global shutdown of translation, transcription, and replication processes is consistent with the stringent response elicited among bacteria during adverse environmental conditions (Srivatsan and Wang, 2008).

Other notable down-regulated genes were linked to the enriched biological processes of nucleotide biosynthesis, including 11 genes involved in the purine or pyrimidine metabolisms (Supplementary Table 4); among them, we found genes encoding the enzymes inosine 5′-monophosphate (IMP) dehydrogenase (GuaB) and GMP synthase (GuaA), two enzymes required for the final two steps of guanine nucleotide biosynthesis from IMP, genes *purM* and *purK*, encoding two enzymes implicated in the synthesis of IMP via *de novo* pathway, and a gene encoding the nucleoside diphosphate kinase (Ndk) of *P. salmonis*, an enzyme that catalyzes the conversion of GDP into GTP. In addition, genes involved in the synthesis of pyrimidine nucleotides, such as *pyrH* and *udk*, were also down-regulated in the intercellular *P. salmonis* (Warner et al., 2014). Down-regulation of nucleotide biosynthesis, particularly GTP biosynthesis, has been associated with the stringent response in Gram-negative and Gram-positive bacteria (Chang et al., 2002; Eriksson et al., 2002; Traxler et al., 2008). The observed down-regulation of nucleotide biosynthetic genes of *P. salmonis* may reflect a decreased need for nucleotides, as ribosome synthesis, a main cellular process that consume nucleotides, is reduced. Additionally, down-regulation of genes involved in the biosynthesis of ATP was also seen during intracellular growth of *P. salmonis*. Four ATP synthase-encoding genes was decreased along with several genes implicated in the glycolytic pathway, including genes encoding terminal enzymes of the pathway, in agreement with a general reduction of metabolic activities (Supplementary Table 4). In addition, three genes encoding subunits of the NADH dehydrogenase operon (*nuoABC*), a complex that functions as the primary aerobic respiratory chain producing ATP via the oxidation of NADH to NAD+, were down-regulated in the intercellular *P. salmonis*. These results suggest that intracellular *P. salmonis* have lower energy requirements and limit their energy production during the infection. Also, they seem consistent with previous studies in *Legionella* showing a (p)ppGpp and starvation-dependent decrease in the relative abundance of RNAs encoding subunits of the ATP synthase complex and genes involved in electron transport chain (Dalebroux et al., 2010; Mendis et al., 2015), suggesting a reduced metabolic activity of the bacterium. Down-regulation of genes involved in major metabolic pathways has been demonstrated for other clinical pathogens as a common response to different starvation conditions, for instance, isoleucine starvation of *E. coli* (Traxler et al., 2008), nutrient starvation of *M. tuberculosis* (Gengenbacher et al., 2010), or glucose starvation of *Streptococcus suis* (Shao et al., 2016).

In different bacterial pathogens, the stringent response is key for activation of survival strategies such as stationary phase, sporulation, biofilm formation, and persistence (Jain et al., 2006). For example, in *L. pneumophila*, the stringent response pathway is important to modulate the virulent attributes that help its survival in the host (Hammer and Swanson, 1999). In fact, a biphasic life cycle has been described in *L. pneumophila*, which relies on the fine-tuning of the levels of (p)ppGpp present in the bacteria. Thus, when nutrients are abundant, bacteria hydrolyze (p)ppGpp and actively multiply and repress the transmission traits (Molofsky and Swanson, 2004; Dalebroux et al., 2009; Trigui et al., 2015). However, as replicating bacteria consume the available nutrients within the vacuolar compartment, (p)ppGpp is produced and accumulated, triggering the entry into the transmissive state that prepare the bacteria to escape from the host cell, to survive in water, and to form a new replicative niche. The role of the stringent response pathway in the pathogenesis of *P. salmonis* is not known yet; however, *P. salmonis* like *Legionella* seems to display both intracellular and extracellular lifestyles; *P. salmonis* not only infects and replicates in its hosts, where it faces starvation, oxidative stress, and other stressors, but it also can survive in seawater for extended periods of time (Fryer and Lannan, 1994) and is transmitted horizontally from fish to fish without the need for physical contact (Rees et al., 2014). Therefore, it is tempting to speculate that differential regulation of the genes that are part of the stringent response pathway may support the survival of the bacteria, and thus the functional analysis of these regulatory pathways would be an interesting area of study in need of further investigation.

### Expression Patterns of Key Stringent Response Genes of *P. salmonis*

**Figure 5:**
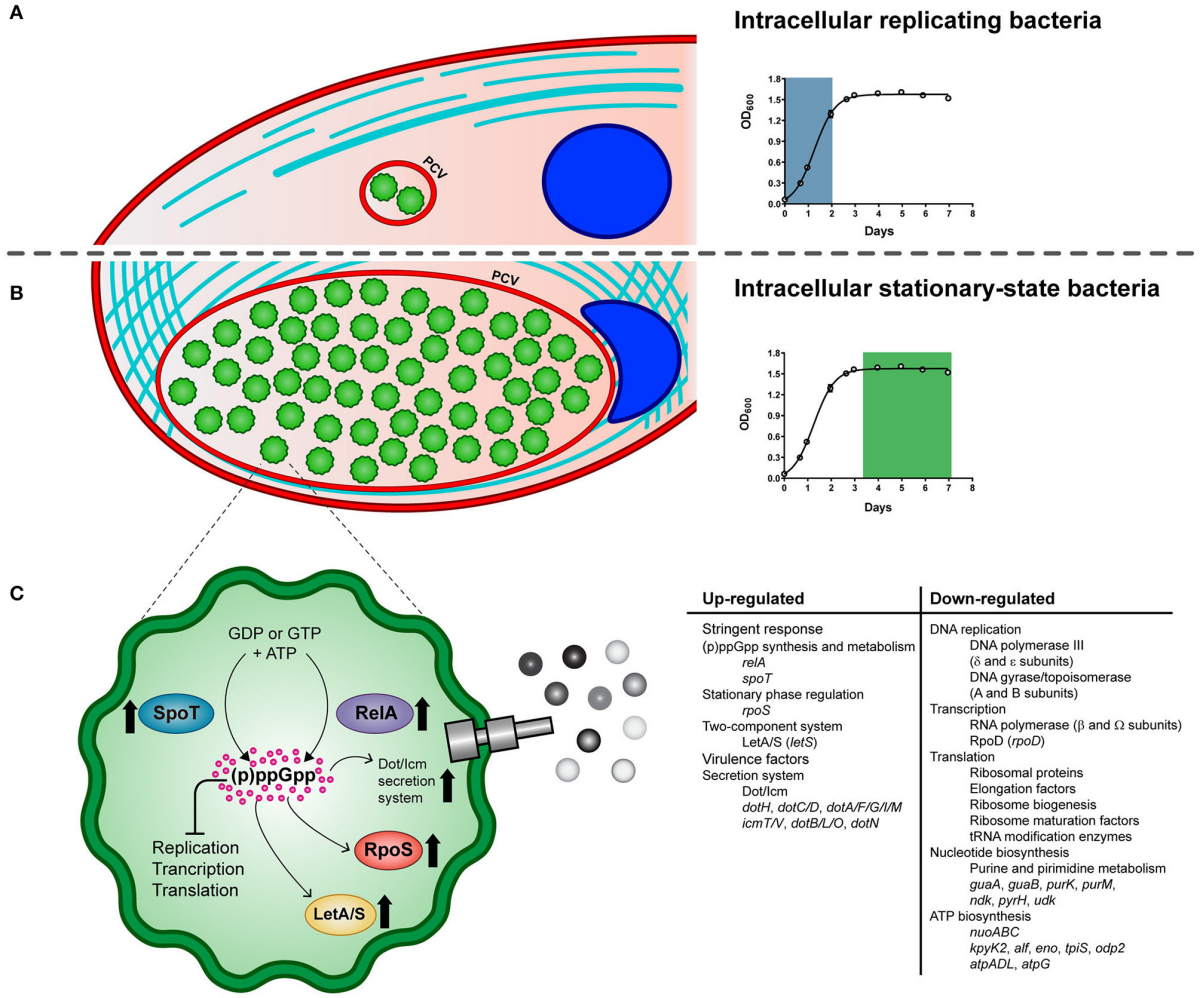
Schematic model of the life cycle of *P. salmonis*. The pathogen alternates between an intracellular replicative phase **(A)** and a stationary phase **(B)**. Entry into stationary phase activates the stringent response of *P. salmonis* **(C)**. It is suggested that amino acid and fatty acid starvation triggers RelA and SpoT to produce the (p)ppGpp. Its accumulation induces the activation of the stress sigma factor RpoS and the LetA/LetS two component system along with a global shutdown of translation, transcription, and replication processes. In addition, several components of the Dot/Icm Type IVB secretion system are induced. PCV: *P. salmonis*-containing vacuole.

It is well-known that in the stationary phase, bacteria activate the stringent response mechanism in order to survive the stressful conditions. Hence, to gain further insights into the stringent response pathway of *P. salmonis*, we compared the gene expression patterns of some key components of the stringent response between intracellular growth and stationary phase and exponential phase of bacterial growth in a rich medium (Table 1, Figure 5). In γ-proteobacteria and β-proteobacteria, (p)ppGpp levels are controlled by two highly conserved enzymes, RelA and SpoT. RelA synthesizes (p)ppGpp in response to amino acid starvation, while SpoT is a bifunctional enzyme, able to synthesize and hydrolyze (p)ppGpp, which modulates (p)ppGpp levels in response to a variety of stresses including fatty acids, phosphate, or iron limitation (Murray and Bremer, 1996; Hauryliuk et al., 2015). Our analysis revealed that *P. salmonis relA* and *spoT* were found to be significantly up-regulated during stationary phase and intracellular growth of *P. salmonis* when compared to exponential growth (Table 1). Similar results have been reported for several pathogens interacting with their host cells, such as *Streptococcus pneumoniae* (Orihuela et al., 2004), *L. pneumophila* (Hammer and Swanson, 1999), *C. burnetii* (Kuley et al., 2015), and *F. tularensis* (Murch et al., 2017). Comparable to well-characterized RelA and SpoT proteins from other bacteria species, predicted *P. salmonis* enzymes consist of two functionally distinct halves, an N-terminal half that includes a hydrolysis (HD) domain, which is inactive in RelA and the (p)ppGpp synthetase domain. The C-terminal half of RelA and SpoT contains TGS and ACT domains, which are involved in mediating inter- and intramolecular interactions and regulating catalytic activity (Atkinson et al., 2011).

**Table 1:** Growth phase and intracellular expression of genes encoding components of the stringent response and the Dot/Icm secretion system.

		**Exponential**		**Stationary**		**Intracellular**	
**Locus**	**ORF**	**Mean**	**SEM**	**Mean**	**SEM**	**Mean**	**SEM**
**Stringent response genes**							
PSLF89_1673	*letS*	38.4	±11.9	450.1^*^	±26.8	**369.7**	±51.7
PSLF89_2849	*spot/rel*	8.1	±1.1	**60.0**	±5.3	**102.8**	±20.9
PSLF89_1546	*relA*	22.3	±2.0	**86.2**	±15.2	**98.7**	±4.1
PSFL89_2050	*rpoS*	6.5	±0.8	**24.9**	±4.2	**42.4**	±1.6
**Icm/Dot type IVB secretion system**							
PSLF89_1866	*IcmK/DotH*	3.6	±0.1	**39.4**	±4.5	**479.4**	±13.5
PSLF89_1867	*IcmE/DotG*	6.9	±0.5	**57.4**	±3.5	**1139.0**	±67.0
PSLF89_1868	*IcmG/DotF*	0.9	±0.03	**18.2**	±0.8	**401.3**	±13.0
PSLF89_1871	*DotD*	15.7	±0.9	**46.6**	±8.3	**8478.6**	±97.3
PSLF89_1873	*DotB*	5.3	±0.5	**45.5**	±3.8	**1004.9**	±60.3
PSLF89_1879	*IcmW*	5.0	±0.3	**119.1**	±11.3	**1788.9**	±83.5
PSLF89_1880	*IcmB/DotO*	6.4	±0.4	**86.0**	±8.6	**1423.4**	±97.5

* *Bold values indicate significant differences (p < 0.05) with respect to exponential phase growth*.

Furthermore, the sigma factor RpoS, which is positively regulated by (p)ppGpp (Oliva et al., 2018), showed the greatest up-regulation (8.5-fold) during intracellular growth of *P. salmonis* and also increased its expression at stationary phase compared to exponential phase growth (Table 1). *P. salmonis* LF89 RpoS shares >70% of amino acid identity with RpoS of *E. coli* and harbors well-conserved Sigma-70 factor regions 1.2, 2, 3, and 4 (Gruber and Gross, 2003). RpoS is a global regulator of gene expression that directs RNA polymerase to transcribe genes important for survival in stationary phase and under other stressful conditions (Dong and Schellhorn, 2010). RpoS modulates the expression of a number of genes including known virulence factors such as the effectors (Hovel-Miner et al., 2009) and the structural components of the T4BSS (Moormeier et al., 2019). In *L. pneumophila*, RpoS is required for intracellular multiplication in amoeba as well as in primary macrophages (Hales and Shuman, 1999; Abu-Zant et al., 2006) and regulates a general silencing of gene expression when *L. pneumophila* is exposed to water (Trigui et al., 2015).

In *L. pneumophila*, the production of (p)ppGpp is followed by the activation of the alternative sigma factor RpoS and the two-component system LetA/LetS (Bachman and Swanson, 2001; Dalebroux et al., 2010), the homolog of GacA/GacS in *Pseudomonas* spp. (Marutani et al., 2008), and UvrY/BarA in *E. coli* (Pernestig et al., 2001). Homologs of LetA/LetS are involved in the virulence phenotypes of several pathogens, among them *E. coli* (Herren et al., 2006), *Pseudomonas syringae* (0Marutani et al., 2008), and *L. pneumophila* (Gal-Mor and Segal, 2003). In several species of Gram-negative bacteria, members of this family of two-component regulators induce the expression of regulatory non-coding RNAs, which sequesters the CsrA repressor, permitting the translation of a number of target mRNAs (Cui et al., 2001; Heeb and Haas, 2001; Molofsky and Swanson, 2003). The homologous genes *letA* and *letS* are encoded in *P. salmonis* LF89 genome and their predicted proteins share >70% and >77% of sequence similarity, respectively, with their counterparts in *E. coli* and *L. pneumophila*. In particular, *letS*, which was found up-regulated during stationary phase and intracellular growth of *P. salmonis* (Table 1), showed the conserved domain architecture of LetS histidine kinases. The N-terminus of *P. salmonis* LetS contains two conserved signal sensing domains: DUF2222 (residues 36–168) and HAMP (residues 191–243), and two transmembrane domains located between residues 10 to 32 and residues 165 to 187. C-terminal to these domains are found the transmitter, receiver, and histidine phosphotransfer domains, which is a characteristic structure of tripartite sensor kinases (Mendis et al., 2018). In *L*. *pneumophila*, LetS acts by shutting down a number of genes related to growth when the bacterium is exposed to water, improving its culturability and survival in a low-nutrient environment (Mendis et al., 2018).

In summary, key genes of the stringent response pathway were up-regulated during *P. salmonis* stationary phase and intracellular growth including genes encoding the two-component sensor kinase LetS, the stationary phase sigma factor RpoS, and the (p)ppGpp synthetase and/or hydrolase RelA and SpoT (Figure 5), which have all been shown to play an important role in regulating virulence in *L. pneumophila* and *C. burnetii* (Oliva et al., 2018; Moormeier et al., 2019).

### Gene Expression Pattern of Predicted Dot/Icm Proteins of *P. salmonis*

We noticed that genes encoding several Dot/Icm proteins were differentially expressed during broth and intracellular growth. Early evidence from *P. salmonis* LF89 genome sequencing revealed putative gene products with high amino acid homology to Dot/Icm proteins of *L. pneumophila* and *C. burnetii* (Gómez et al., 2013; Pulgar et al., 2015b; Cortés et al., 2017). The majority of *P. salmonis dot*/*icm* genes were found on three regions of the chromosome, with the *icmF* and *dotU* genes lying outside of three main regions. In this work, we found that 15 genes encoding structural components of the Dot/Icm proteins were significantly up-regulated during intracellular growth, including 10 out of 12 genes that conform one of the three *P. salmonis dot*/*icm* gene clusters. Up-regulated genes encoded the outer membrane (OM) protein DotH, OM lipoproteins DotC/D, inner membrane (IM) proteins DotA/F/G/I/M and IcmT/V, IM ATPases DotB/L/O, and the cytosolic proteins DotN. No homologs of the T4BSS effector molecules reported for *L. pneumophila* and *C. burnetii* were found in the genome of *P. salmonis*; however, a number of hypothetical genes found interspersed within the *dot*/*icm* clusters could be potential effector molecule candidates or a unique part of the secretion machinery. Here, we sought to associate T4BSS function with *P. salmonis* growth phase and virulence by monitoring the expression of genes encoding a structural component of the secretion apparatus and one hypothetical protein located within the gene cluster. qPCR analysis revealed that the entire set of genes was significantly up-regulated during stationary phase and intracellular growth of the bacteria when compared with the exponential phase of growth (Table 1). These data suggest that *P. salmonis* could be actively modifying its vacuole via T4BSS at this late stage of infection; this situation is unlike that reported for *L. pneumophila*, where type T4BSS is required only coincident with and immediately following infection for establishment of a replicative vacuole (Segal et al., 2005). However, the pattern of expression of these components of the *P. salmonis* T4BSS may provide some clues to its function. For example, our observation that maximal *P. salmonis* cytopathogenicity is reached in the stationary phase suggests that these *dot*/*icm* components mediate escape from the host cell or, alternatively, they may be required during *P. salmonis* uptake to develop a new replication vacuole.

In summary, our results suggest that *P. salmonis* shares with several other bacterial pathogens the ability to respond to changing conditions to ensure its survival in the environment. A detailed understanding of the regulation of *P. salmonis* virulence will likely facilitate the identification of the effector functions that enable this pathogen to parasitize eukaryotic cells. Knowledge of the *P. salmonis* virulence regulation machinery may also suggest methods to eradicate this pathogen from the environment or the infected fish.

## Data Availability Statement

The datasets generated for this study can be found in the Gene Expression Omnibus. Accession number: GSE128825.

## Author Contributions

AZ carried out microarray hybridization and immunofluorescence studies. PA designed and performed cell viability and cytotoxicity assays and genome equivalent measurements. DT and RP performed the bioinformatics analyses during genome annotation for microarray construction and transcriptomic studies. JO-S carried out characterization of bacterial growth and infection process and development of RNA extraction protocols. AM, FC, and MG contributed to data analysis and discussion, as well as during the preparation of the manuscript. VC oversaw the entire work and wrote most parts of the manuscript.

### Conflict of Interest

The authors declare that the research was conducted in the absence of any commercial or financial relationships that could be construed as a potential conflict of interest.
