# Supplementary material for: Transcriptomic Changes of Piscirickettsia salmonis During Intracellular Growth in a Salmon Macrophage-Like Cell Line
Source: Front Cell Infect Microbiol. 2020 Jan 9;9:426. doi: 10.3389/fcimb.2019.00426 (PMC6964531; doi:10.3389/fcimb.2019.00426)
Supplement: Supplementary file 1 [file Image_1.pdf]

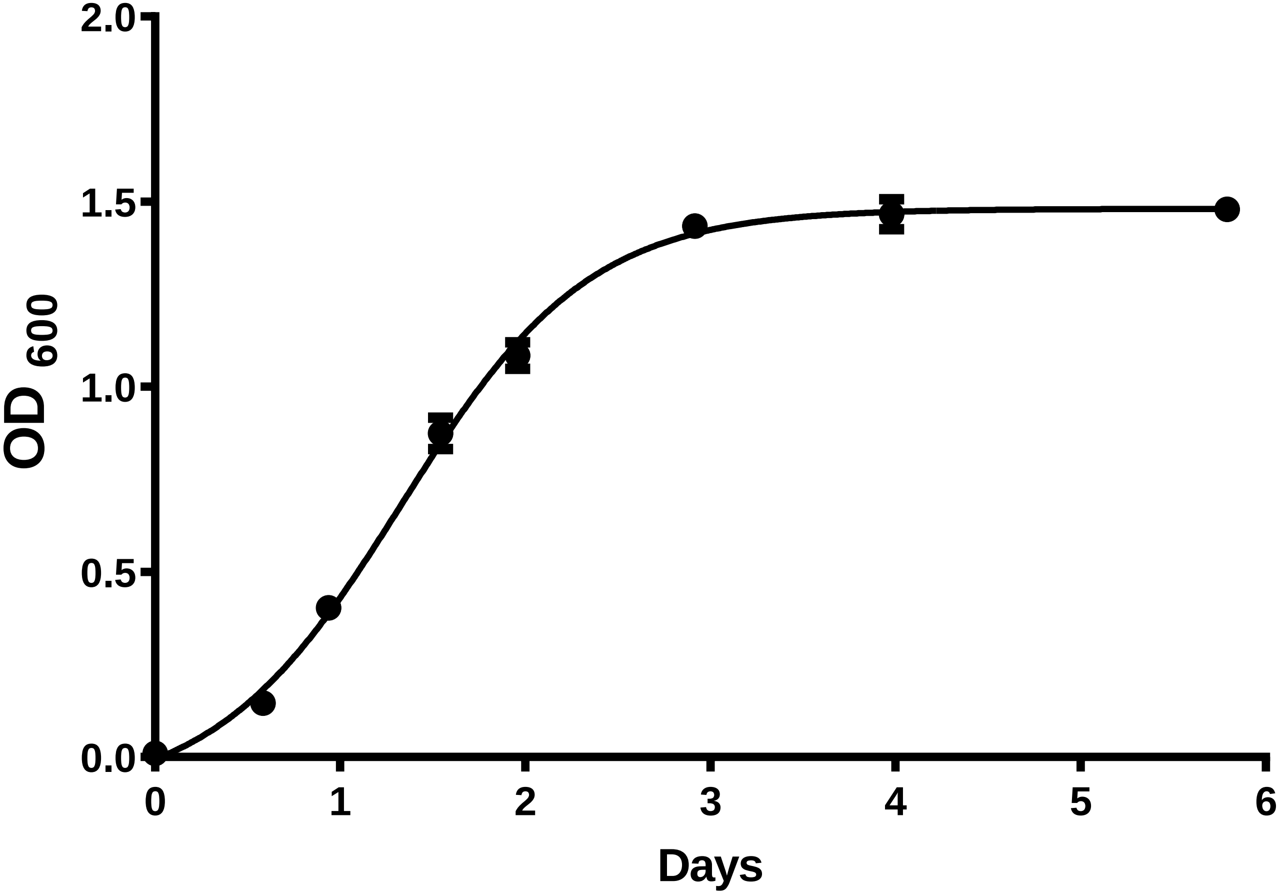

Supplementary Figure 1. Growth curve of *P. salmonis* LF-89. Bacterial growth in a rich culture media was evaluated by measuring the optical density of the cultures at 600 nm (OD_{600}) for six days (n=4 ±SD).
