# Supplementary material for: Transcriptomic Changes of Piscirickettsia salmonis During Intracellular Growth in a Salmon Macrophage-Like Cell Line
Source: Front Cell Infect Microbiol. 2020 Jan 9;9:426. doi: 10.3389/fcimb.2019.00426 (PMC6964531; doi:10.3389/fcimb.2019.00426)
Supplement: Supplementary file 2 [file Image_2.pdf]

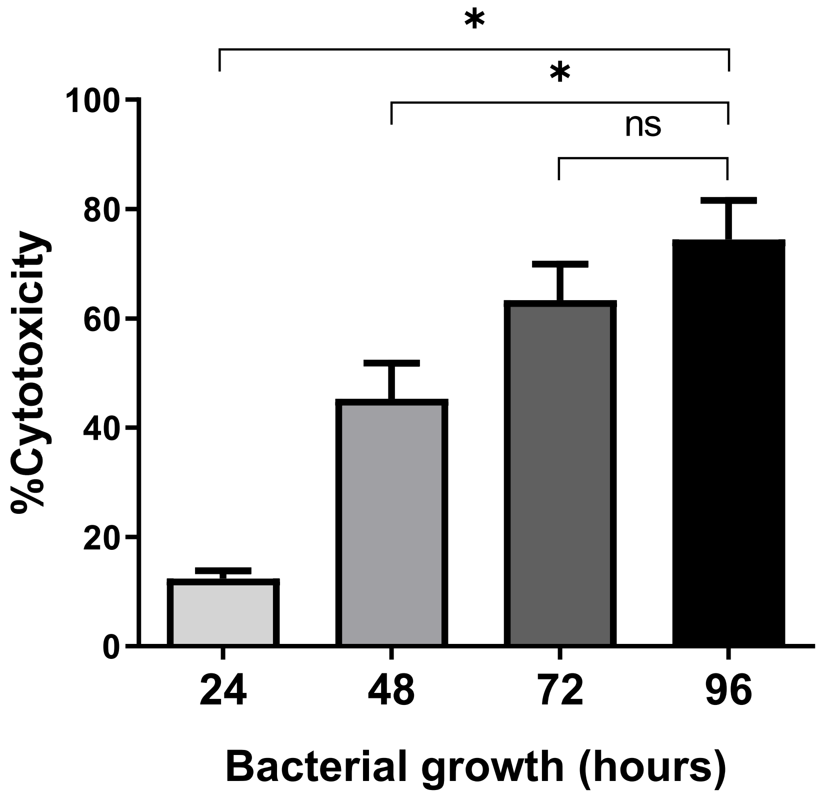

Supplementary Figure 2. Cytotoxic effect of *P. salmonis* to SHK-1 cells. The capacity of viable macrophages to reduce the alamarBlue colorimetric reagent was quantified 10 days after incubating the cells with bacteria grown for 24, 48, 72 or 96 hours in liquid broth. Data reflect means ± SD (N=12 biological replicates), asterisks above the bars indicate significant differences ($p<0.05$). Data were analyzed with GraphPad Prism V8.0.1 using an unpaired t-test with Welch`s correction.
