# Supplementary material for: Transcriptomic Changes of Piscirickettsia salmonis During Intracellular Growth in a Salmon Macrophage-Like Cell Line
Source: Front Cell Infect Microbiol. 2020 Jan 9;9:426. doi: 10.3389/fcimb.2019.00426 (PMC6964531; doi:10.3389/fcimb.2019.00426)
Supplement: Supplementary file 3 [file Image_3.pdf]

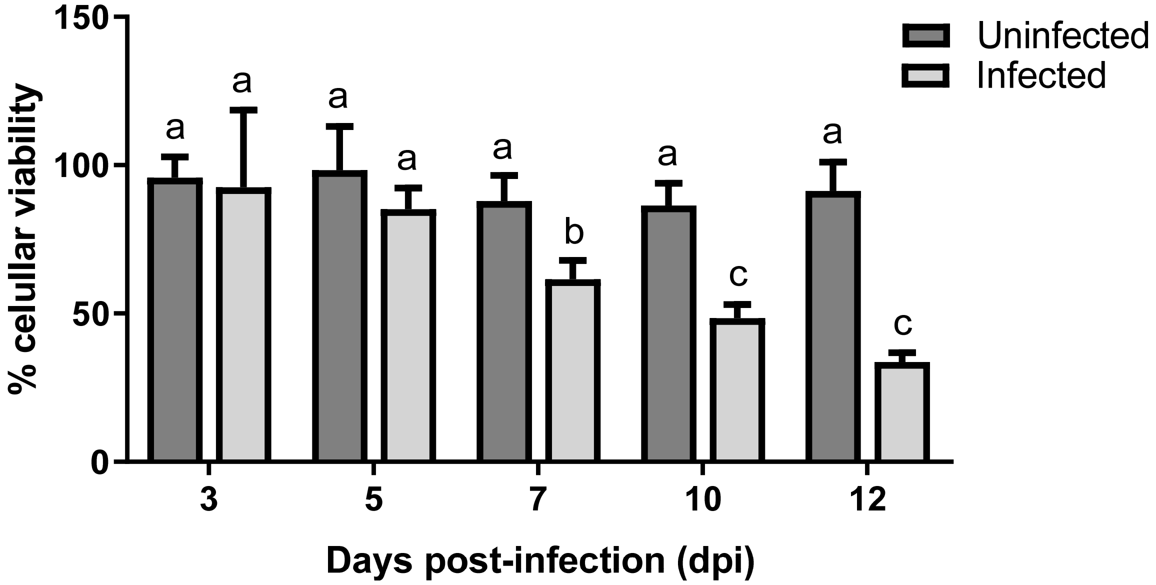

Supplementary Figure 3. Effect of *P. salmonis* infection on the viability of SHK-1 cells. After three-day incubation period with the bacteria, cells were washed with fresh media and cell viability was monitored at different days post-infection with the alamarBlue method as described in Materials and Methods. The percentages of alamarBlue reduction were calculated with respect to cells at the beginning of the assay. The reduction in viability of the SHK-1 cells infected with *P. salmonis* reached approximately 30% of control's viability at 12 dpi. Data reflect means ± SD (N=10 biological replicates). Distinct letters mean statistically significant differences within groups (two-way ANOVA followed by followed by Tukey's test, $p<0.001$).
