# Supplementary figures and images for: Transcriptomic Changes of Piscirickettsia salmonis During Intracellular Growth in a Salmon Macrophage-Like Cell Line
Source: Front Cell Infect Microbiol. 2020 Jan 9;9:426. doi: 10.3389/fcimb.2019.00426 (PMC6964531; doi:10.3389/fcimb.2019.00426)

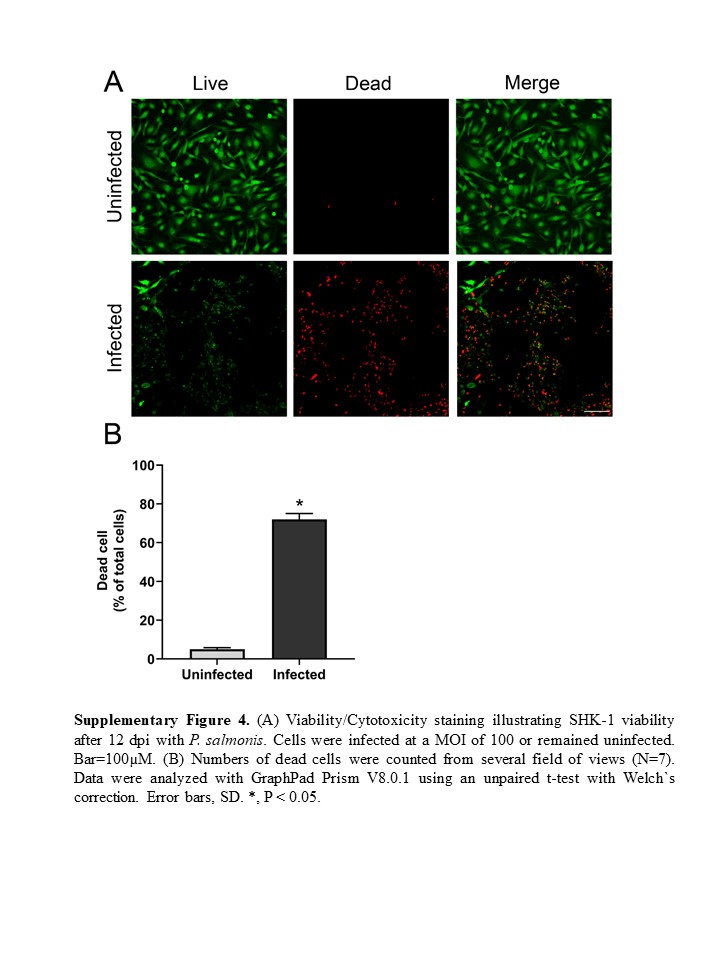

Supplement: Supplementary file 4 [file Image_4.jpeg]
