# Supplementary material for: Transcriptomic Changes of Piscirickettsia salmonis During Intracellular Growth in a Salmon Macrophage-Like Cell Line
Source: Front Cell Infect Microbiol. 2020 Jan 9;9:426. doi: 10.3389/fcimb.2019.00426 (PMC6964531; doi:10.3389/fcimb.2019.00426)
Supplement: Supplementary file 5 [file Image_5.pdf]

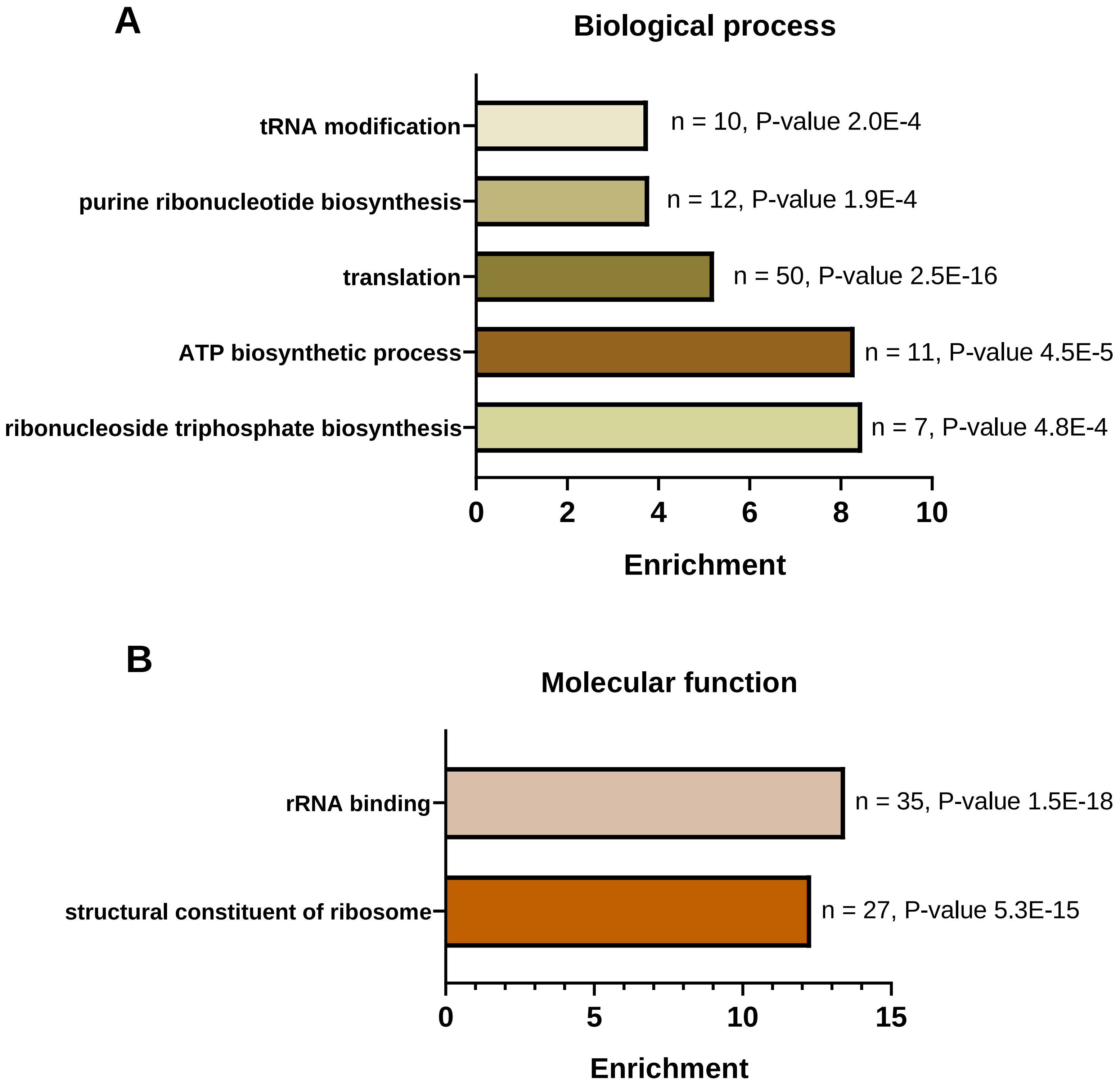

Supplementary Figure 5. Gene ontology analysis. Bar graphs show the enriched GO terms of biological process (A) and molecular functions (B). Results are shown only for most specific GOs, i.e. with the lowest level in the GO directed acyclic graph. $p < 0.01$ was considered statistically significant.
