# Supplementary material for: Transcriptomic Changes of Piscirickettsia salmonis During Intracellular Growth in a Salmon Macrophage-Like Cell Line
Source: Front Cell Infect Microbiol. 2020 Jan 9;9:426. doi: 10.3389/fcimb.2019.00426 (PMC6964531; doi:10.3389/fcimb.2019.00426)
Supplement: Supplementary file 6 [file Table_1.pdf]

Supplementary Table 1. Correlation between stationary phase bacterial cultures, optical density measurements, bacterial cell number and bacterial viability. *P. salmonis* cultures grown for 2 or 4 days were used to measure optical density at 600 nm (OD_{600}), bacterial cell numbers were counted with a Petroff-Hausser chamber, and bacteria viability was evaluated with the most-probable number (MPN) method. The average of four independent experiments with their respective standard deviation is shown.

Days of culture	OD_{600}	Bacterial cell number	MPN
4	1.26 ±0.06	5.78 ±0.25 x 10^9	1.03 ±0.33 x 10^7
2	0.57±0.08	1.70 ±0.12 x 10^9	4.90 ±0.31 x 10^6
