# Supplementary material for: Transcriptomic Changes of Piscirickettsia salmonis During Intracellular Growth in a Salmon Macrophage-Like Cell Line
Source: Front Cell Infect Microbiol. 2020 Jan 9;9:426. doi: 10.3389/fcimb.2019.00426 (PMC6964531; doi:10.3389/fcimb.2019.00426)
Supplement: Supplementary file 7 [file Table_2.pdf]

Supplementary Table 2: List of genes and primer sequences used in this study.

Locus	Gene annotation	Primer sequence 5` → 3`	
PSLF89_3276	Alternative sigma factor RpoS	Forward<br>Reverse	CAGGAAGTCCAGATGCAACA<br>CTTTCTATCATCCGCCGTCT
PSLF89_2050	Alternative sigma factor RpoS	Forward<br>Reverse	CAACCTTGATGCGACAGAGA<br>ACGGCGAGCAAAAATATCAC
PSLF89_1673	Histidine kinase GacS	Forward<br>Reverse	AAGCAGGTGCTGCAGGTTAT<br>CTGCGCAACATCAGGACTAA
PSLF89_3272	Histidine kinase KdpD	Forward<br>Reverse	CCGTTAGCACGTCTTGGTTT<br>TTTGACAAAACGCTCAGCAC
PSLF89_2952	Peptidyl-prolyl cis-trans isomerase Mip	Forward<br>Reverse	TGGTGTAGTTCGCCATTTGA<br>ATAAGCAAGCTTGGCTGGAA
PSLF89_1p169	Phosphatidylserine PLD	Forward<br>Reverse	ATGTGCAATGGCTATGCTGA<br>ATCAGGCCAACTTTCCAATG
PSLF89_3295	Protein-tyrosine-phosphatase	Forward<br>Reverse	TTAGCGCTGAGCTGATCAAA<br>CTTCATTACGCCACCTACCC
PSLF89_34	Zn-dependent metalloprotease HpaT	Forward<br>Reverse	AGAAAATGGTGAGCCAATGC<br>GACCTGCGACATCCAAAGAT
PSLF89_2849	(p)ppGpp synthase/hydrolase Spot	Forward<br>Reverse	ATGCTGGACGCGGTATTATC<br>GACATTTCCACACGAACACG
PSLF89_1546	(p)ppGpp synthase/hydrolase RelA	Forward<br>Reverse	CCAAGTGGTCTGGCAATTTT<br>CCTCAGTGCGTAAAACGTGA
PSLF89_1160	Glucose-6-phosphate isomerase	Forward<br>Reverse	CGGCACAAATCAATCATCAC<br>ACCCAGTCCCACATTGGTAA
PSLF89_1860	Gluconate kinase	Forward<br>Reverse	ATCCAGCGCTACCTAAAGCA<br>GTTGCAATTGCGCTGAAGTA
PSLF89_1979	Exoribonuclease R Rnr	Forward<br>Reverse	TCGCGTTGTTGAGATTCTTG<br>CTTGTTCTTGCACAGCCTCA
PSLF89_839	Selenocysteine lyase/Cysteine desulfurase Csd	Forward<br>Reverse	CATGTTGGCATTTGCTATGG<br>CGCTGCAATCATACCTGCTA
PSLF89_2324	3'5'-cyclic AMP phosphodiesterase CpdA	Forward	CTACCCATGGTCGCTTTGTT

		Reverse	TTGGGTCAATCTGCATGGTA
PSLF89_386	Disulfide bond formation protein DsbB	Forward<br>Reverse	CTGCAACACTACCCCCTGTT<br>CCAGGTGGCTAAGCTGATGT
PSLF89_1195	Transcriptional accessory protein Tex/SPT6	Forward<br>Reverse	CCTTGAAGGGGTAGTCACCA<br>ATGTCACCCGCTTTAACGAC
PSLF89_2576	DNA-binding response regulator QseB	Forward<br>Reverse	GAGAGCGAGCCTGTCAATCT<br>CAACCGTAGAGGGATTGCAT
PSLF89_913	Stringent starvation protein B SspB	Forward<br>Reverse	GGGTTTGGTGGTGTTTTACG<br>GGGCAATATCACGACCATCT
PSLF89_609	DotC	Forward<br>Reverse	CACCTCCTACTTGGCGTGAT<br>GCCCTTCGGTCAACAATTTA
PSLF89_1866	DotH	Forward<br>Reverse	CCGTGCAGGTCTGGTCTTAT<br>AGAAGGCGTTGCTGTCAACT
PSLF89_1867	DotG	Forward<br>Reverse	ATGCCAAATTGTTGGGTAGC<br>TTTTGTGCTGTTGTGGCATT
PSLF89_1868	DotF	Forward<br>Reverse	AAGGCCGATGTTGCATTATT<br>GCCTTTCGCTGACTTACTGC
PSLF89_1869	DotD	Forward<br>Reverse	GGCAACGGGGTTTTATTTTT<br>TCTCCGCTAACATCACCACA
PSLF89_1871	DotD	Forward<br>Reverse	CGGTTCAACAAACAGGGACT<br>CCCACAGCCTTTGCAATATC
PSLF89_1873	DotB	Forward<br>Reverse	CAGTCGTGAGCCAAAGTGAA<br>ATAACCGCCTCAAGTGTTGG
PSLF89_1874	IcmT	Forward<br>Reverse	GCTTGATGCACGTGTCTGTT<br>TCGCGCAGGACAATAAAAA
PSLF89_1875	DotL	Forward<br>Reverse	TTCAATGTGGTCATCGCCTA<br>TTTACCACTTCCGGTTGAGC
PSLF89_1877	DotM	Forward<br>Reverse	TCGACGACCCATGATATGAA<br>TTTCTTCACCAACCCAAACC
PSLF89_1879	IcmW	Forward<br>Reverse	CAACAAAGGTTGCCGAGGTA<br>TGAGCTGATGCAGCAAGTAAA
PSLF89_1880	DotO	Forward<br>Reverse	GATATTGGGCCTTCAAGCAA<br>GCCCCTAACTGCGTATCAAA
PSLF89_1884	DotI	Forward	ATGGCTGGGAGCAGTATTTG

		Reverse	TTTGAGTATCTCGCGAAGCA
PSLF89_3032	Glycosyltransferase WaaE	Forward<br>Reverse	GTGAAGGTTTCGGCGTATGT<br>CCAGAATCCGCGACAATAAT
PSLF89_3128	LPS assembly outer membrane protein LptD	Forward<br>Reverse	ACGATCATGCCCACACTGTA<br>GTTTGGCGTAAATTGCGTTT
PSLF89_2434	Outer membrane protein OmpA	Forward<br>Reverse	TTCACCTACAGTGGCAGCAG<br>CCATTGTCGAACCCATATCC
PSLF89_1754	Predicted arabinose efflux Bcr	Forward<br>Reverse	TGACTGCGGAGAGAGGTTTT<br>AGCCACCCTTATCAAACACG
PSLF89_908	Fe2+ transport system protein B FeoB	Forward<br>Reverse	ATGCGGCGAACTTAGAAAGA<br>GATCGGTGCCTTGGTTTTTA
PSLF89_1995	Periplasmic protein TonB	Forward<br>Reverse	CATAAAGGAACGCAACGTGA<br>TTCAGCATTTGTCGAACAGC
PSLF89_1992	Biopolymer transport protein ExbB/TolQ	Forward<br>Reverse	GGGCACCATTAAACATCCAG<br>AAGGACTGTGCCCAACAAAC
PSLF89_1p227	Uncharacterized protein contains pentapeptide repeats	Forward<br>Reverse	TGGGTGGAATCTTGAAGGAG<br>TTTTTCGCAACAGTGCCTAA
PSLF89_1876	Hypothetical protein	Forward<br>Reverse	CACGTATGCCAGTTCGATGT<br>AGCCGATTCGTAAGAGCAAA
PSLF89_1881	Hypothetical protein	Forward<br>Reverse	CTGTGATTTCAAGCGTGAGC<br>TGACTGCGGAGAGAGGTTTT
PSLF89_1120	Hypothetical protein	Forward<br>Reverse	GTGAATGGATGTCGGGCTAT<br>AGCCTTCAGTCCAAGCGTTA
PSLF89_1p229	Hypothetical protein	Forward<br>Reverse	TGAAGACCGTGGATACACACA<br>GGGAAGAGGTTGAAGAGTCG
PSLF89_2654	Hypothetical protein	Forward<br>Reverse	CGGTCGTCCAAAACTTCCTA<br>TTTTCAAAGCAGAACGAGCA
PSLF89_751	Hypothetical protein	Forward<br>Reverse	TATGCCTACACCAGGGCATT<br>AAGTTTCGCGGCATATCATT
PSLF89_08290	Hypothetical protein	Forward<br>Reverse	CGCGATTGTTAGGACCTGTT<br>AATGGGGAATGATACGCACT
