# Supplementary material for: Transcriptomic Changes of Piscirickettsia salmonis During Intracellular Growth in a Salmon Macrophage-Like Cell Line
Source: Front Cell Infect Microbiol. 2020 Jan 9;9:426. doi: 10.3389/fcimb.2019.00426 (PMC6964531; doi:10.3389/fcimb.2019.00426)
Supplement: Supplementary file 9 [file Table_4.pdf]

Supplementary Table 4. Significantly enriched biological process among down-regulated genes.

Translation		
**Locus**	**Gene name**	**Description**
PSLF89_683	RSMI	16S rRNA C1402 (ribose-2'-O) methylase RsmI
PSLF89_49	RSME	16S rRNA U1498 N3-methylase RsmE
PSLF89_1974	HFLX	50S ribosomal subunit-associated GTPase HflX
PSLF89_2444	QUEC	7-cyano-7-deazaguanine synthase
PSLF89_69	FMT	Methionyl-tRNA formyltransferase
PSLF89_09040		mRNA-degrading endonuclease
PSLF89_68	DEF1	Peptide deformylase
PSLF89_277	PNP	Polyribonucleotide nucleotidyltransferase
PSLF89_2891	RS1	Polyribonucleotide nucleotidyltransferase
PSLF89_1761	TGT	Queuine/archaeosine tRNA-ribosyltransferase
PSLF89_2005	RIMM	Ribosomal 30S subunit maturation factor RimM
PSLF89_358	RL14	Ribosomal protein L14
PSLF89_372	RL17	Ribosomal protein L17
PSLF89_363	RL18	Ribosomal protein L18
PSLF89_2007	RL19	Ribosomal protein L19
PSLF89_353	RL2	Ribosomal protein L2
PSLF89_945	RL20	Ribosomal protein L20
PSLF89_07680	RL22	Ribosomal protein L22
PSLF89_352	RL23	Ribosomal protein L23
PSLF89_07670	RL24	Ribosomal protein L24
PSLF89_3005	RL25	Ribosomal protein L25
PSLF89_2320	RL27	Ribosomal protein L27
PSLF89_350	RL3	Ribosomal protein L3
PSLF89_07665	RL30	Ribosomal protein L30/L7E
PSLF89_235	RL31	Ribosomal protein L31
PSLF89_207	RL33	Ribosomal protein L33
PSLF89_03395	RL34	Ribosomal protein L34
PSLF89_351	RL4	Ribosomal protein L4
PSLF89_359	RL5	Ribosomal protein L5
PSLF89_362	RL6	Ribosomal protein L6P/L9E
PSLF89_2633	RL9	Ribosomal protein L9
PSLF89_349	RS10	Ribosomal protein S10
PSLF89_345	RS12	Ribosomal protein S12
PSLF89_368	RS13	Ribosomal protein S13
PSLF89_360	RS14	Ribosomal protein S14
PSLF89_276	RS15	Ribosomal protein S15P/S13E

PSLF89_2004	RS16	Ribosomal protein S16
PSLF89_2634	RS18	Ribosomal protein S18
PSLF89_354	RS19	Ribosomal protein S19
PSLF89_1238	RS2	Ribosomal protein S2
PSLF89_984	RS20	Ribosomal protein S20
PSLF89_355	RS3	Ribosomal protein S3
PSLF89_06495	RS6	Ribosomal protein S6
PSLF89_346	RS7	Ribosomal protein S7
PSLF89_361	RS8	Ribosomal protein S8
PSLF89_911	RS9	Ribosomal protein S9
PSLF89_311	RATA	Ribosome association toxin PasT (RatA)
PSLF89_271	RIMP	Ribosome maturation factor RimP
PSLF89_1241	RRF	Ribosome recycling factor
PSLF89_274	RBFA	Ribosome-binding factor A
PSLF89_720	ROC4	RNA recognition motif (RRM) domain
PSLF89_03400	RNPA	RNase P protein component
PSLF89_1769	SYS	Seryl-tRNA synthetase
PSLF89_3144	YBEY	ssRNA-specific RNase YbeY 16S rRNA maturation enzyme
PSLF89_942	SYT	Threonyl-tRNA synthetase
PSLF89_347	EFG	Translation elongation factor EF-G a GTPase
PSLF89_1239	EFTS	Translation elongation factor EF-Ts
PSLF89_348	EFTU	Translation elongation factor EF-Tu a GTPase
PSLF89_925	EFP	Translation elongation factor P (EF-P)
PSLF89_04980	IF1	Translation initiation factor IF-1
PSLF89_2815	TSAB	tRNA A37 threonylcarbamoyladenosine modification protein TsaB
PSLF89_1567	TRMJ	tRNA C32U32 (ribose-2'-O)-methylase TrmJ or a related methyltransferase
PSLF89_705	TRMH	tRNA G18 (ribose-2'-O)-methylase SpoU
PSLF89_2006	TRMD	tRNA G37 N-methylase TrmD
PSLF89_2563	MNMA	tRNA U34 2-thiouridine synthase
PSLF89_275	TRUB	tRNA U55 pseudouridine synthase
PSLF89_385	TRML	tRNA(Leu) C34 or U34 (ribose-2'-O)-methylase
PSLF89_947	SYFB	tRNA-binding EMAP/Myf domain

tRNA modification enzymes

Locus	Gene name	Description
PSLF89_69	FMT	methionyl-tRNA formyltransferase
PSLF89_07620	TRMB	methyltransferase
PSLF89_1302	TADA	adenosine deaminase

PSLF89_1761	TGT	queuine tRNA-ribosyltransferase
PSLF89_2006	TRMD	tRNA (guanine-N1)-methyltransferase
PSLF89_2563	MNMA	tRNA-specific 2-thiouridylase
PSLF89_275	TRUB	pseudouridine synthase
PSLF89_2815	TSAB	tRNA threonylcarbamoyladenosine
PSLF89_385	TRML	rRNA methylase
PSLF89_705	TRMH	tRNA guanosine-2'-O-methyltransferase

Transcription/replication

Locus	Gene name	Description
**PSLF89_2848**	RPOZ	DNA-directed RNA polymerase subunit K/omega
**PSLF89_3276**	RPOH	DNA-directed RNA polymerase sigma subunit (sigma70/sigma32)
**PSLF89_343**	RPOB	DNA-directed RNA polymerase beta subunit/140 kD subunit
**PSLF89_2177**	DPO3E	DNA polymerase III epsilon subunit or related 3'-5' exonuclease
**PSLF89_794**	HOLB	DNA polymerase III delta prime subunit
**PSLF89_4**	GYRB	DNA gyrase/topoisomerase IV subunit B
**PSLF89_2894**	GYRA	DNA gyrase/topoisomerase IV subunit A

Purine/pyrimidine metabolism

Locus	Gene name	Description
PSLF89_1300	GUAB	inosine 5'-monophosphate dehydrogenase
PSLF89_1301	GUAA	GMP synthase
PSLF89_1570	NDK	nucleoside diphosphate kinase
PSLF89_2968	PURM	Phosphoribosylaminoimidazole (AIR) synthetase
PSLF89_3319	PURK	Phosphoribosylaminoimidazole carboxylase (NCAIR synthetase)
PSLF89_3321	ORFM	non-canonical purine NTP pyrophosphatase
	DEOD	purine nucleoside phosphorylase
PSLF89_1240	PYRH	UMP kinase
PSLF89_2141	UDK	uridine kinase

Biosynthesis of ATP

Locus	Gene name	Description
PSLF89_1026	PGK	3-phosphoglycerate kinase
PSLF89_1027	KPYK2	Pyruvate kinase
PSLF89_1028	ALF	Fructose/tagatose bisphosphate aldolase
PSLF89_2045	ENO	Enolase
PSLF89_253	TPIS	Triosephosphate isomerase

PSLF89_3189	ODP2	Pyruvate/2-oxoglutarate dehydrogenase complex
PSLF89_3382	ATPA	FoF1-type ATP synthase alpha subunit
PSLF89_3383	ATPD	FoF1-type ATP synthase delta subunit
PSLF89_3385	ATPL	FoF1-type ATP synthase membrane subunit c/Archaeal/vacuolar-type H+-ATPase subunit K
PSLF89_3386	ATP6	FoF1-type ATP synthase membrane subunit a
PSLF89_256	NUOA	NADH:ubiquinone oxidoreductase subunit 3 (chain A)
PSLF89_258	NUOC	NADH:ubiquinone oxidoreductase 27 kD subunit (chain C)
PSLF89_257	NUOB	NADH:ubiquinone oxidoreductase 20 kD subunit (chain B)
